# The association between impairment of HDL cholesterol efflux capacity and atrial remodeling in atrial fibrillation

**DOI:** 10.1038/s41598-021-82998-4

**Published:** 2021-02-11

**Authors:** Asuka Minami-Takano, Hiroshi Iwata, Katsutoshi Miyosawa, Tomoyuki Shiozawa, Hidemori Hayashi, Takehiro Funamizu, Kai Ishii, Yui Nozaki, Haruna Tabuchi, Gaku Sekita, Kazunori Shimada, Masataka Sumiyoshi, Yuji Nakazato, Hiroyuki Daida, Tohru Minamino

**Affiliations:** 1 Department of Cardiovascular Biology and Medicine, Juntendo University Graduate School of Medicine, Tokyo, Japan; 2 Department of Clinical Engineering, Juntendo University Hospital, Hongo 2-1-1, Bunkyo, Tokyo 113-0033 Japan; 3 Tokyo New Drug Research Laboratories, Kowa Company, Ltd, Tokyo, Japan; 4 Department of Cardiovascular Medicine, Juntendo University Nerima Hospital, Tokyo, Japan; 5 Department of Cardiovascular Medicine, Juntendo University Urayasu Hospital, Chiba, Japan; 6 Japan Agency for Medical Research and Development-Core Research for Evolutionary Medical Science and Technology (AMED-CREST), Japan Agency for Medical Research and Development, Tokyo, Japan

**Keywords:** Cardiology, Cardiovascular biology, Cardiac regeneration

## Abstract

This cross-sectional study enrolled 202 patients with atrial fibrillation (AF) who had undergone catheter ablation and evaluated the association between high-density lipoprotein (HDL) functionality, cholesterol efflux capacity (CEC) of HDL, and the pathophysiology of left atrial structural remodeling. Participants were divided into two groups, based on their left atrial volume index (LAVI) (< 34 mL/m^2^, n = 60 vs. LAVI ≥ 34 mL/m^2^, n = 142). We quantified three types of HDL CECs by the presence or absence of cyclic-AMP, as entire, and CEC dependent or not dependent on ATP binding cassette transporter A1 (ABCA1) and termed them Global CEC, ABCA1 CEC, and Non-ABCA1 CEC, respectively. Consequently, Global and Non-ABCA1 CECs were significantly impaired in patients with an enlarged LA (Global CEC: p = 0.039, Non-ABCA1 CEC: p = 0.022). Logistic regression analyses demonstrated that Non-ABCA1 CEC was significantly associated with an enlarged LA after adjusting for the conventional risk factors of AF. Furthermore, the association of higher Non-ABCA1 CEC with an enlarged LA was independent of serum levels of HDL cholesterol and serum myeloperoxidase (Odds ratio of 1 standard deviation higher: 0.64, 95% confidence interval: 0.43–0.95, p = 0.027). The findings of this study indicate the potential contribution of reduced Non-ABCA1 CEC in HDL to the pathophysiology in left atrial structural remodeling of patients with AF.

## Introduction

Structural remodeling in the left atrium (LA) represented by 3-dimensional (3D) expansion with fibrotic changes has been considered to be a central pathological process in the development and progression of AF. However, its complicated mechanisms remain largely obscure. This study explores the mechanistic insights of progressive atrial structural remodeling in AF, which is globally increasing^1^ and is associated with elevated risks of heart failure, embolic events and death^2–4^.

The association of an elevated serum high-density lipoprotein (HDL) cholesterol (HDL-C) level with a reduced risk of atherosclerotic cardiovascular events has been known for more than 40 years^5^. However, the failure of HDL-C raising therapies, including niacin^6^ and cholesteryl-ester transfer protein (CETP) inhibitors^7^, to show their prognostic benefit in line with the evidence of genetic studies indicating adverse effects of very high HDL-C levels^8^ has challenged the long-standing HDL-hypothesis. The differences between the quantity of HDL, serum HDL-C level, and the quality of HDL, such as its functionality, the abundance of particles and the distribution of subspecies, may have caused the discrepancy between the HDL-hypothesis and recent results observed in clinical trials of HDL-C raising therapies^9^. Therefore, the significance of HDL functionality, defined by the capacity to take up excessive cholesterol from macrophages in the local tissue, has been gathering intense clinical attention due to its possible direct anti-atherogenic properties leading to favorable outcomes in atherosclerotic cardiovascular disease^10^.

Previous studies have indicated that atherosclerotic cardiovascular disease and AF share common pathological mechanisms, such as low-grade long-lasting chronic inflammation^11^, excess oxidative stress^12^, and clinical risk factors, such as age, hypertension, smoking habits and diabetes^13^. However, the association between HDL functionality and the pathogenesis of AF has not been adequately evaluated. Therefore, we conducted this cross-sectional study in AF patients to investigate the relationship between the extent of the progression in LA structural remodeling and HDL functionality, which was measured in terms of three types of cholesterol efflux capacity (CEC), ie, Global CEC, ATP binding cassette transporter A1 (ABCA1) mediated CEC, and Non-ABCA1 mediated (Non-ABCA1) CEC.

## Results

### Baseline patient demographics in groups with and without large LAVI: advanced structural remodeling of LA

The baseline demographics of patients with or without advanced LA remodeling are compared in Table 1. The large left atrial volume index (LAVI) group, defined as LAVI ≥ 34 mL/m^2^, included more than twice the number of patients (n = 142, 70.3%) compared to the normal LAVI group, LAVI < 34 mL/m^2^ (n = 60, 29.7%). The prevalence of hypertension was higher in the large LAVI group, while there were no differences in other complications of AF between the groups, such as diabetes and chronic kidney disease. Patients with a large LAVI had higher CHADS_2_ and CHA_2_DS_2_-VASc scores, and a higher serum N-terminal pro b-type natriuretic peptide (NT-proBNP) level. Serum levels of high sensitivity C-reactive protein (hs-CRP) were similar in both groups. The prevalence of persistent AF was higher in the large LAVI group, indicating that patients in the large LAVI group had a more advanced stage of AF. Moreover, left ventricular ejection fraction (LVEF) was lower in the large LAVI group compared to that in the normal LAVI group. With respect to medications, the prevalence of patients taking angiotensin converting enzyme inhibitors (ACEIs) or angiotensin receptor blockers (ARBs) was higher in the large LAVI group, although the incidences for anti-arrhythmic drugs, beta blockers, calcium channel blockers, and statins were similar. Furthermore, no significant differences in lipid parameters, total cholesterol, LDL-C, HDL-C, triglyceride and apolipoprotein levels, or ApoA1 and ApoB100 were observed between the groups.

**Table 1:** Background demographics of patients with or without advanced LA remodeling.

	LAVI < 34 mL/m^2^	LAVI ≥ 34 mL/m^2^	p value
	(n = 60)	(n = 142)	
Age, year	60.8 ± 8.2	62.6 ± 8.6	0.16
Gender male, n (%)	41 (68.3)	105 (73.9)	0.49
BMI, kg/m^2^	24.8 ± 4.1	25.1 ± 3.5	0.58
Hypertension, n (%)	**19 (32.2)**	**69 (49.3)**	**0.029**
Dyslipidemia, n (%)	30 (50.9)	77 (55.4)	0.64
Diabetes mellitus, n (%)	5 (8.5)	25 (18.0)	0.13
Chronic kidney disease, n (%)	7 (11.9)	23 (16.4)	0.51
Smoking history, n (%)	28 (47.5)	88 (62.9)	0.059
CHADS_2_ score	**0 (0–1)**	**1 (0–1)**	** < 0.001**
CHA_2_DS_2_-VASc score	**1 (0–2)**	**2 (1–2)**	**0.003**
AF duration, month	12.0 (4.0–34.5)	17.0 (6.0–48.0)	0.15
NT-proBNP (pg/mL)	82.5 (53.4–533.2)	330.6 (163.9–745.3)	** < 0.001**
hs-CRP (mg/L)	0.05 (0.03–0.10)	0.06 (0.03–0.13)	0.18
**Atrial fibrillation type**			
Paroxymal	**48 (80.0)**	**73 (51.4)**	** < 0.001**
Persistent	**12 (20.0)**	**69 (48.6)**	
**Lipid profiles**			
Total Cholesterol (mg/dL)	198.5 ± 28.8	193.3 ± 33.3	0.30
LDL-C (mg/dL)	117.2 ± 25.6	115.5 ± 28.2	0.57
HDL-C (mg/dL)	55.8 ± 12.2	52.5 ± 12.6	0.093
Triglyceride (mg/dL)	108.0 (80.0–155.0)	117.0 (87.5–154.0)	0.37
ApoB100 (mg/dL)	95.4 ± 19.9	94.4 ± 19.6	0.73
ApoA1 (mg/dL)	144.1 ± 22.7	139.0 ± 22.8	0.14
**Echocardiography**			
LVEF (%)	**67.0 ± 6.5**	**63.0 ± 8.9**	**0.002**
Left atrial diameter (LAD) (mm)	**35.7 ± 4.3**	**41.8 ± 5.0**	** < 0.001**
Left atrial volume index (LAVI) mL/m^2^	**28.3 ± 4.2**	**45.3 ± 11.0**	** < 0.001**
**Medications**			
Anti-arrhythmic drugs, n (%)	39 (66.1)	98 (70.0)	0.61
Beta-blockers, n (%)	29 (49.2)	75 (53.6)	0.64
ACE-I or ARB, n (%)	**7 (11.9)**	**46 (32.9)**	**0.003**
CCB, n (%)	12 (20.3)	35 (25.0)	0.58
Statins, n (%)	13 (22.0)	32 (22.9)	1

ACE-I indicates angiotensin-converting enzyme inhibitor; ARB, angiotensin receptor blocker; ApoA1, apolipoprotein A1; ApoB100, apolipoprotein B100; BMI, body mass index; HDL-C, high-density lipoprotein cholesterol; hs-CRP, high-sensitivity C-reactive protein; LDL-C, low-density lipoprotein cholesterol; LVEF, left ventricular ejection fraction; and NT-proBNP, N-terminal pro-brain natriuretic peptide.

### Compromised cholesterol efflux capacity, CEC index, in AF patients with enlarged LAVI

**Figure 1:**
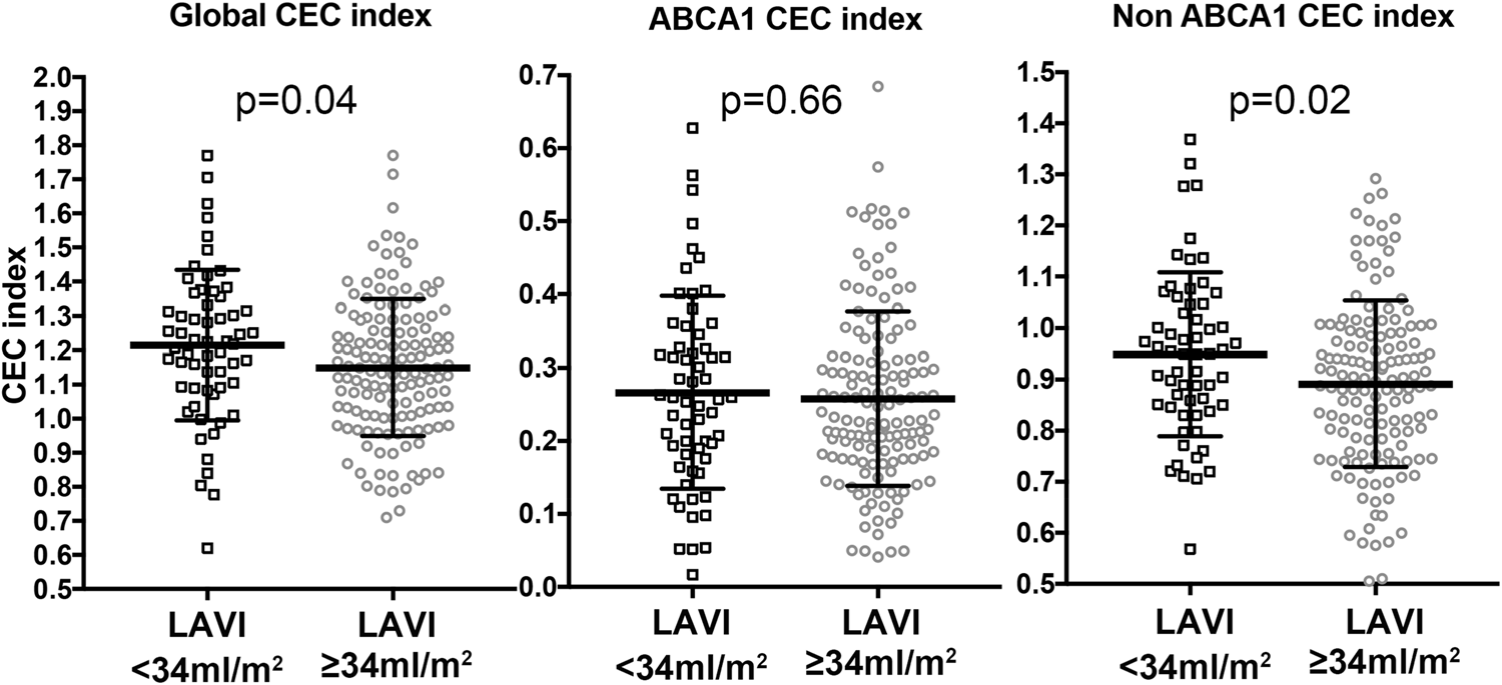
Global CEC, ABCA1 CEC and Non-ABCA1 CEC in patients with and without an enlarged left atrium (LAVI ≥ and < 34 mL/m^2^). Global CEC, ABCA1 CEC and non-ABCA1 CEC were measured in AF patients with LAVI ≥ and < 34 mL/m^2^. The value was normalized with the respective QC value on each assay plate to calculate the indexed value. Each open square/circle represents one participant. The horizontal lines indicate average ± standard deviation, respectively.

Although there were no significant differences in lipid parameters, the Global as well as Non-ABCA1 CEC indexes were significantly attenuated in the large LAVI group. In contrast, the ABCA1 CEC index, which was calculated by subtracting Non-ABCA1 CEC from Global CEC, was similar in both groups (Global: 1.21 ± 0.22% vs 1.15 ± 0.20%: p = 0.039, ABCA1: 0.27 ± 0.13% vs 0.26 ± 0.12%: p = 0.66, Non-ABCA1: 0.95 ± 0.16% vs 0.89 ± 0.16%: p = 0.022, respectively, Fig. 1). However, no linear correlation was found using Spearman’s test between any CEC index and LAVI in the entire patient population or in patients with and without enlarged LA. Moreover, to evaluate the HDL functionality in distinct types of AF regarding its clinical progression, the participants were divided into paroxysmal and persistent AF (PAF, n = 121, 59.9% vs. PeAF, n = 81, 40.1%), and then we compared the three types of CEC indexes. Consequently, Non-ABCA1 CEC was significantly compromised in patients with persistent AF compared with those with paroxysmal AF (Supplementary Fig. 1). Moreover, in sex- and age-matched AF and non-AF populations (n = 41 in each group) by propensity score matching, Global and Non-ABCA1 CEC indexes were significantly impaired in AF patients compared to Non-AF patients, who underwent catheter ablation for supraventricular tachycardia (SVT) (n = 27, 65.8%) and ventricular premature contraction (VPC)/ ventricular tachycardia (VT) (n = 14, 34.2%) (Supplementary Fig.2).

### Reduced HDL2-cholesterol (HDL2-C) level in patients with large LAVI

**Figure 2:**
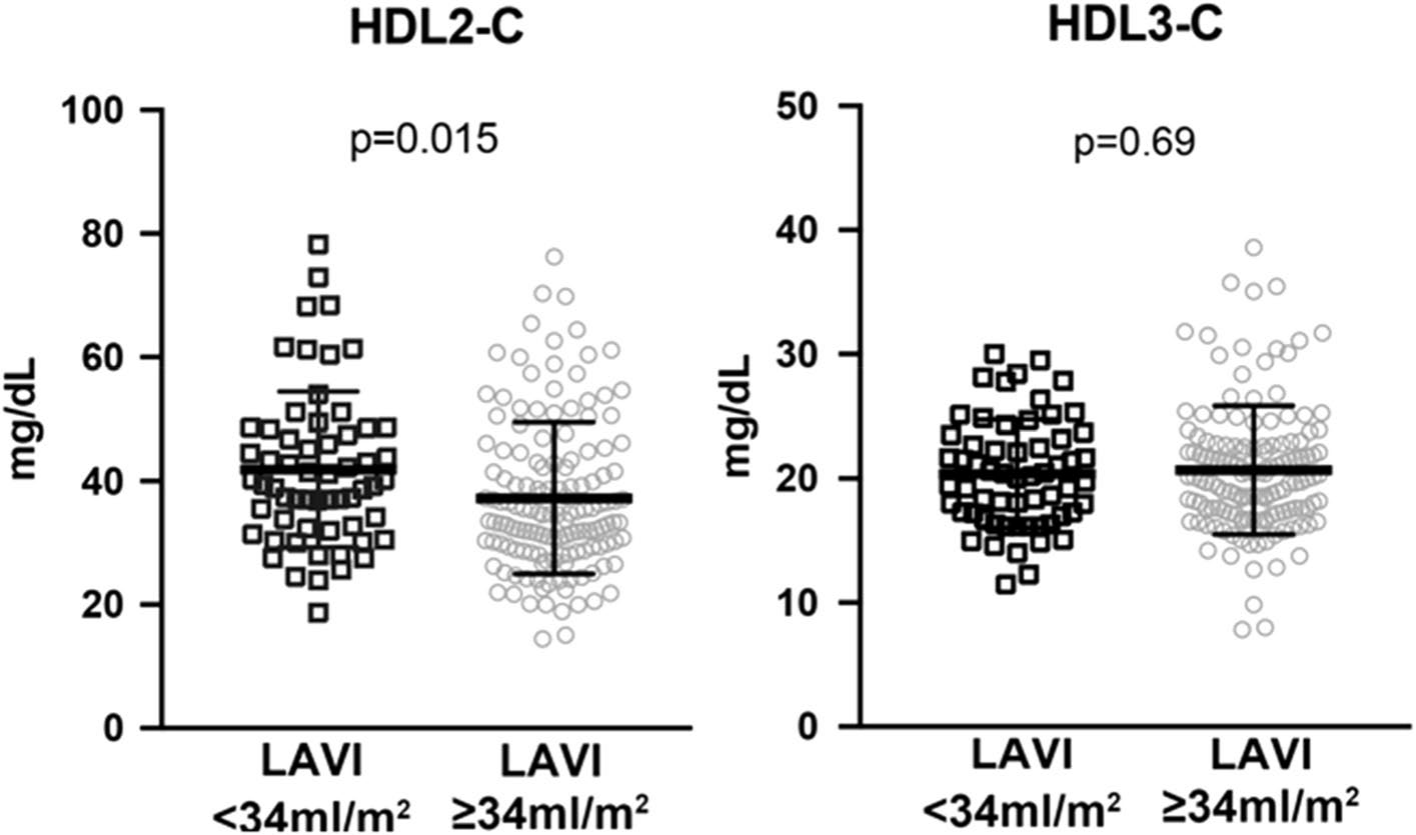
Plasma high-density lipoprotein2 and 3 -cholesterol level (HDL-2C and 3C) in patients with and without an enlarged left atrium (LAVI ≥ and < 34 mL/m^2^). Subfractions of HDL, HDL2 and HDL3, were separated by ultracentrifugation and then cholesterol concentration was measured. Each open square/circle represents one participant. The horizontal lines indicate average ± standard deviation, respectively.

In order to further elucidate the mechanistic insights of the association between HDL functionality and LA enlargement in AF patients, we measured the two major subfractions of HDL-C, HDL2-cholesterol (HDL2-C) and HDL3-cholesterol (HDL3-C) levels, by their density using ultracentrifugation^14^. We found the HDL2-C was significantly lower in patients with large LAVI (HDL2-C: 41.9 ± 12.6 mg/dL vs 37.2 ± 12.2 mg/dL: p = 0.015). In contrast, HDL3-C was similar in both groups. (HDL3-C: 20.4 ± 4.4 mg/dL vs 20.7 ± 5.2 mg/dL: p = 0.694) (Fig. 2).

### Higher serum myeloperoxidase (MPO) levels in patients with large LAVI

**Figure 3:**
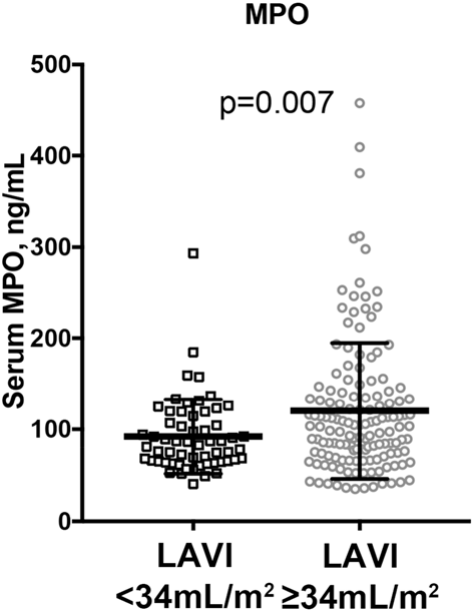
Serum levels of myeloperoxidase (MPO) in patients with and without an enlarged left atrium (LAVI ≥ and < 34 mL/m^2^). Serum MPO levels were measured by ELISA. Each open square/circle represents one participant. The horizontal lines indicate average ± standard deviation, respectively.

Previous evidence has indicated that MPO, a proinflammatory, pro-oxidative and pro-fibrotic enzyme, is significantly associated not only with the pathogenesis of atherosclerotic cardiovascular disease^15,16^, but also with that of AF^17^. Moreover, MPO has been also demonstrated to mediate oxidative modification of Apolipoprotein A1 (ApoA1), leading to dysfunctional HDL in which ABCA1 CEC is impaired^18^. Based on these findings, we measured the serum level of MPO in our study subjects. As expected, the serum MPO level was significantly higher in the large LAVI group (92.1 ± 40.7 ng/mL vs 120.3 ± 74.4 ng/mL: p = 0.0067, Fig. 3). Furthermore, there was no correlation between serum MPO level and any of the three types of CEC indexes (Correlation coefficient (r) and *p* value (p) of serum MPO vs. Global CEC index: r = 0.0037, *p* = 0.96, vs. ABCA1 CEC index: r = 0.013, *p* = 0.86, and vs. Non-ABCA1 CEC index: r = 0.0068,* p* = 0.92, respectively). Similarly, another inflammatory indicator, serum amyloid A (SAA), which was previously shown to be associated with impaired SR-B1 mediated (Non-ABCA1) CEC^19^, was measured. However, in contrast to MPO, levels of SAA in patients with and without enlarged LA were similar (Supplementary Fig. 3) and no significant correlation was found in SAA levels with any CEC index (vs. Global CEC index: r = -0.036, *p* = 0.67, vs. ABCA1 CEC index: r = -0.03, *p* = 0.70, vs. Non-ABCA1 CEC index: r = -0.035, *p* = 0.65). While MPO and SAA were significantly correlated with hs-CRP (hs CRP vs. MPO: r = 0.19, *p* = 0.009 and vs. SAA r = 0.41, *p* < 0.001), respectively, there was no correlation between MPO and SAA (r = 0.065, *p* = 0.40) (Supplementary Fig. 4).

**Figure 4:**
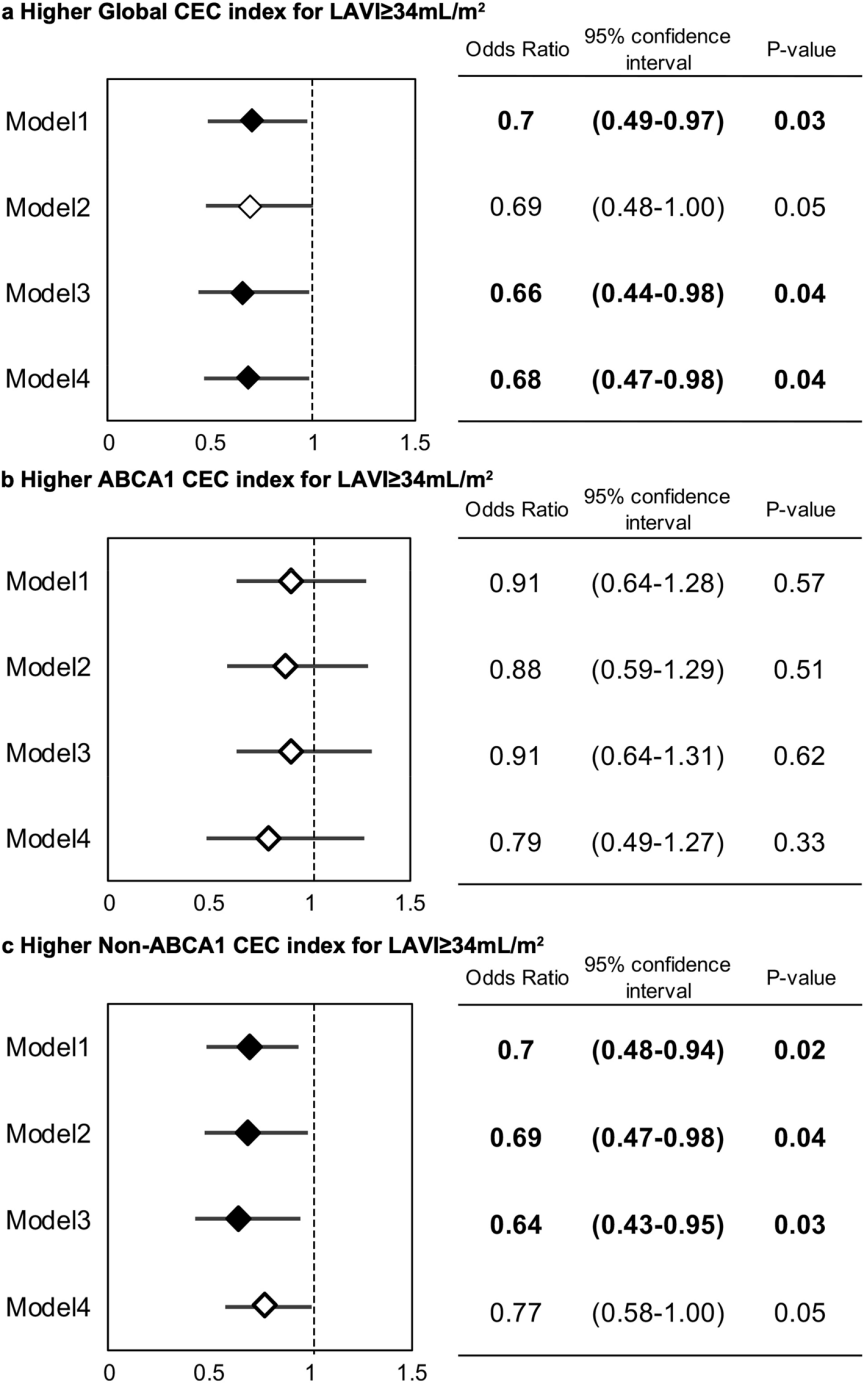
Odds ratios of 1 standard deviation (1SD) higher Global, ABCA1, and Non-ABCA1 cholesterol efflux capacity (CEC) indexes for an enlarged left atrium. Adjusted multivariate logistic regression analyses using 3 models assessed the odds ratios of 1SD higher of Global CEC (a), ABCA1 CEC (b) and Non-ABCA1 CEC (c) indexes and LAVI ≥ 34 mL/m^2^. Model 1 included age, male gender, hypertension, smoking history, and 1SD higher left ventricular ejection fraction (LVEF). Model 2 included the covariates of Model 1 and 1 higher logarithmic NT-proBNP (Log_10_ NT-proBNP) instead of 1SD higher of LVEF. Model 3 included the serum level of high density lipoprotein cholesterol (HDL-C) and serum myeloperoxidase (MPO) in addition to those in Model 1. Model 4 included all previously established risk factors for the progression of the atrial structural remodeling in AF patients available in the present study, including age, male gender, hypertension, diabetes, smoking history, and biomarkers of heart failure (NT-proBNP), inflammation (hs-CRP) and obesity (BMI). Any CECs significantly associated with LAVI ≥ 34 mL/m^2^ are shown as closed rhombuses, while open rhombuses are CECs without statistical significance.

### Significant associations of large LAVI with Global CEC and Non-ABCA1 CEC indexes of HDL, but no association with ABCA1 CEC index

Multiple logistic regression analyses were conducted to assess the significance of the association between the three types of HDL-CEC and the progression of atrial structural remodeling in AF patients in which LAVI ≥ 34 mL/m^2^ was chosen as the objective variable. In the three models used in the adjusted multivariate analyses, explanatory covariates assessed with the three types of CECs were identified in accordance with the results of the univariate analyses (Supplementary Table1) in addition to age and gender, which were included in all models. In the univariate and multivariate logistic regression analyses, age, body mass index (BMI), duration of AF, plasma level of NT-proBNP, serum level of high-sensitivity C reactive protein (hs-CRP), LVEF, and serum levels of lipid parameters and MPO were assessed as continuous variables. History of hypertension, smoking history, 1 higher logarithmic NT-proBNP (Log_10_ NT-proBNP), 1 standard deviation (1SD) higher LVEF, and 1 higher Log_10_ MPO were significantly associated with LAVI ≥ 34 mL/m^2^. Moreover, there were significant inverse associations between 1SD higher Global CEC, as well as Non-ABCA1 CEC indexes and large LAVI, while there was no association with ABCA1 CEC index.

Consistent with the univariate analyses, 1SD higher Global CEC and Non-ABCA1 CEC were continuously and independently associated with large LAVI through the three different models (Fig. 4a,c, Supplementary Table 2a, c), while the ABCA1 CEC index was continuously demonstrated not to be associated (Fig. 4b, Supplementary Table 2b). In particular, the associations in the Global CEC and Non-ABCA1 CEC indexes were significant even after adjustment for serum levels of MPO and HDL-C (Model 3), indicating that higher Global and Non-ABCA1 CECs of HDL and serum HDL-C or MPO level were statistically independent with respect to the association with a large LAVI. Conversely, none of the univariate and multivariate analyses adjusted by the three types of CEC indexes demonstrated a significant association of serum HDL-C level with a large LAVI, while that of MPO was continuously and significantly associated.

## Discussion

The findings of the present study have demonstrated that Global CEC and Non-ABCA1 CEC of HDL were impaired in AF patients with advanced structural remodeling of LA, which was represented by a large LAVI, compared to those with a normal LAVI. Similarly, in patients with persistent AF, Non-ABCA1 CEC was significantly impaired compared to that in patients with paroxysmal AF. In subfractions of HDL-C, a large spherical particle of HDL, HDL2-C, was significantly lower in patients with large LAVI, while there was no difference in HDL3-C in the two groups. Univariate and multivariate logistic regression analyses revealed that elevated indexes of Global CEC as well as Non-ABCA1 CEC, but not ABCA1 CEC, were inversely associated with a large LAVI in AF patients, which was independent from plasma NT-proBNP, LVEF, serum HDL-C, and MPO. These findings indicate a potential pathophysiology of the impairment of cholesterol efflux by HDL in left atrial enlargement, structural remodeling, and the pathogenesis of AF. Moreover, our findings in this study suggest that Non-ABCA1-mediated cholesterol efflux might be important in the pathogenesis of AF among its multiple pathways.

Most of the previously established risk factors of AF, such as hypertension, obesity, diabetes, dyslipidemia, and smoking history, are shared by those of atherosclerotic cardiovascular disease (ASCVD)^20^. However, the overlap pathophysiology of these two common and clinically relevant cardiovascular disorders has not been fully clarified. In this study, the prevalences of conventional atherosclerotic risk factors, including hypertension and smoking history, were significantly higher in patients with an enlarged LA, and presumably represent an advanced stage of AF, while those of diabetes and chronic kidney disease were similar in both groups.

Recent clinical studies have been shedding light on HDL-C as a possible contributor in the pathogenesis of AF by demonstrating that a low serum HDL-C level was associated with an increased risk of the development and progression of AF^21^. However, in this study, even though there were slightly reduced HDL-C levels in patients with an enlarged LA, univariate and multivariate logistic regression analyses demonstrated no association between the serum level of HDL-C and a large LAVI. In contrast, the association between Global CEC and Non-ABCA1 CEC of HDL and enlarged LA was independent from serum HDL-C, which was consistent with numerous lines of evidence suggesting the significance of HDL functionality, rather than serum HDL-C levels, for the pathogenesis and prognosis of various cardiovascular diseases ^10,22,23^. A recent study has demonstrated that the CEC of HDL was lower in patients with AF compared to those of the non-AF control cohort^24^. However, there is a paucity of data regarding the association between AF progression and different cholesterol efflux pathways, as cholesterol efflux is facilitated by either ABCA1-mediated or non-ABCA1 mediated pathways^25^. This study showed a significant and consistent impairment of Non-ABCA1 mediated CEC in AF patients with advanced left atrial structural remodeling, while no significant impact of ABCA1 mediated CEC, which added some new insight into the impact of transporter-specific CEC of HDL in AF pathogenesis. Moreover, the HDL2-C level was significantly lower in patients with large LAVI compared to normal LAVI, while there was no difference in HDL3-C. These findings might be consistent with findings that Non-ABCA1 dependent CEC of HDL was significantly impaired in patients with advanced LA remodeling in the present study, as cholesterol efflux of HDL2, which is a mature and larger spherical subspecies of HDL, has been demonstrated to be dominantly mediated via non ABCA1 pathway, such as scavenger receptor BI (SR-BI) and ATP Binding Cassette Subfamily G Member 1 (ABCG1)^26^. In addition, the progression of atrial 3D structural remodeling was represented by LA volume index in this study, which was superior in the precise measurement of 3D expansion of LA and exhibited less operator-dependent variance compared with simple 2D LA diameter measurement^27^.

Accumulating evidence has indicated that the MPO induced oxidative modification of HDL impairs the cholesterol efflux capacity of HDL^28^, particularly the ABCA1-mediated cholesterol efflux^18^, although inconsistent results have been reported^29^. Atheroprotective HDL is transformed into dysfunctional HDL by inflammation and oxidative stress^18^, both of which have been demonstrated to be critical in both structural and electrical remodeling of the atrium in AF patients^30^. Previous studies indicated that MPO acts not only as a pro-inflammatory and pro-oxidant enzyme in ASCVD, but also as a pro-fibrotic mediator in the pathogenesis of AF progression^11,17^. In this study, patients in the large LAVI group had significantly higher serum MPO levels, while levels of hs-CRP, a marker of systemic inflammation, were similar in both groups. Moreover, univariate and adjusted multivariate logistic regression analyses showed that a higher preprocedural serum MPO level was significantly associated with an enlarged LA, while that of hs-CRP was not. As previous reports have shown that MPO impairs particularly ABCA1 CEC of HDL by oxidative modification^31^, we have postulated there is an inverse correlation of serum MPO with ABCA1 CEC of HDL. However, it was not correlated with any type of CEC. Therefore, the results in this study may suggest the dominant contribution of Non ABCA1-mediated CEC, including that via SR-BI^32^ and ABCG1^33^, to atrial structural remodeling in AF. Taken together, in the pathogenesis of AF, the contributions of elevated MPO and the impairment of CEC of HDL to the pathological structural remodeling of the atrium might be independent, and not through the impairment of ABCA1 CEC. Moreover, the results in the present study that there was no correlation between MPO and any CEC of HDL pathways were comparable with those of a previous study^29^, indicating no substantial influence of oxidative impairment in any pathways of CEC by MPO on AF pathophysiology.

This study has several limitations. First, it employed a cross-sectional approach with a relatively small-scale cohort involving 202 Japanese patients in a single center registry, therefore, it is necessary to confirm these findings in larger scale and multicenter studies. Second, parameters which have been indicated to be associated with HDL function, such as the number of HDL-particles^34^, paraoxonase activity^35^, and cholesterol ester transfer protein activity^36^, might have had a pathological effect on the progression of LA structural remodeling. Thus, in future studies, these parameters may need to be evaluated with respect to their relationship with the development and progression of AF. Third, lifestyle measures other than smoking habit which have an effect on the pathogenesis in AF, including alcohol consumption, were not available in the present study. Finally, we collected only preprocedural blood samples before the catheter ablation procedure. Considering the recent report that compromised CEC in AF returned to normal after the restoration of sinus rhythm by catheter ablation^24^, changes in CEC over multiple time points may be warranted. Even with these limitations, the strengths of the present study include a detailed evaluation of the pathological role of impairment of multiple types of CEC, such as Global, ABCA1 dependent, and Non-ABCA1 dependent CEC, in the progression of atrial structural remodeling, which was precisely indicated by LAVI. Therefore, the findings in this study may have clinical implications for CEC, Non-ABCA1 CEC in particular, for structural atrial remodeling in AF. Therefore, the present findings may represent further evidence regarding the pathological roles of impaired HDL functionality in AF.

In conclusion, impairment of Non-ABCA1 mediated CEC of HDL was associated with the progression of 3D-structural atrial remodeling in AF. In-depth analysis of HDL functionality in this study provides evidence which can assist with the development of therapeutic strategies for preventing disease advancement in AF by improving HDL functionality.

## Methods

### Study participants and study design

This was a cross-sectional observational cohort analysis enrolling consecutive 261 patients with AF, between the ages of 40 and 75, who had undergone catheter ablation between July 2017 and January 2019 at Juntendo University Hospital, Tokyo, Japan. Left atrial volume index (LAVI), measured by 2-dimensional echocardiography, was used to represent the extent of 3D-structural remodeling, since LAVI describes structural remodeling better than LA diameter^27^. Individuals whose LAVI data were unavailable were excluded from analysis (n = 59) so 202 patients were included in the analysis (Supplementary Fig. 5. In accordance with the recommendations of the European Association of Cardiovascular Imaging (EACVI) and European Heart Rhythm Association (EHRA) Expert Consensus Document^37^, the patients were divided into two groups, those with or without advanced structural LA remodeling based on LAVI < or ≥ 34 mL/m^2^. This study protocol was approved by the Institutional Review Board of Juntendo University School of Medicine and publicly registered (UMIN000041762). All participants provided written informed consent before their participation in this study according to the guidelines established in the Declaration of Helsinki.

### Blood samples and biochemical measurements

Venous blood for standard blood tests was obtained on the day of catheter ablation before the procedure. To measure the CEC of HDL and serum myeloperoxidase (MPO) levels, samples were collected through the femoral vein via blood access for the procedure. Within 30 min after blood collection, the serum was separated by centrifugation (1,700 × g for 10 min) and then stored at -80 °C until use. Serum MPO level was measured with a human MPO ELISA kit (Human Myeloperoxidase DuoSet ELISA, R&D Systems, Minneapolis, MN) according to the manufacturer’s protocol. Serum SAA levels were measured by latex immunoturbidimetric assay at a commercially available external laboratory (SRL Inc, Tokyo, Japan).

### Assessment of atrial structural remodeling in AF, left atrial volume index (LAVI)

Two-dimensional echocardiographic images of the left ventricle (LV) and left atrium (LA) were obtained by the parasternal long axis and apical two- and four-chamber views with second harmonic imaging using standard methods. The echocardiographic parameters were obtained on the basis of the American Society of Echocardiography guidelines^38^ by experienced sonographers. Left atrium (LA) volume was calculated by two-dimensional echocardiographic images and normalized by body surface area (BSA) and represented as LAVI^27^ using the biplane Simpson method.

### Measurement of three types of cholesterol efflux capacity by HDL

To assess the association between the pathophysiology in AF and HDL functionality, we measured three types of HDL CECs by using serum of patients with AF who were about to undergo catheter ablation. Global CEC, ABCA1 CEC, and Non-ABCA1 CEC of HDL were measured using methods previously described with modifications^39^. Apolipoprotein B (apoB)-depleted serum was prepared by adding 40 μL of 20% polyethylene glycol 6000 (Sigma-Aldrich, St. Louis, MI, USA) in 200 mM glycine (pH 7.4) to 100 μL of serum and then centrifugation at 8,000 × g for 30 min. The supernatants were stored at -80 °C until use. J774.1 murine macrophage-like cells, obtained from Japanese Collection of Research Bioresources (JCRB) Cell Bank (Osaka, Japan), were seeded in 48-well plates at 75,000 cells per well and then labeled with [^3^H]-cholesterol (1 μCi/mL, PerkinElmer, Waltham, MA, USA) for 24 h at 37 °C in RPMI1640 medium (Thermo Fisher Scientific, Waltham, MA, USA) supplemented with 5% fetal bovine serum (FBS), acyl-CoA:cholesterol acyltransferase (ACAT) inhibitor Sandoze 58–035 (2 μg/mL, Sigma-Aldrich), and 50 μg/mL acetylated LDL (Ac-LDL, Alfa Aesar, Ward Hill, MA, USA) to enrich the cells with free cholesterol. The cells were equilibrated for 24 h in RPMI1640 medium supplemented with 0.2% bovine serum albumin (Roche, Basel, Switzerland) and 2 μg/mL ACAT inhibitor in the presence (Global CEC) or absence (Non-ATP-binding cassette transporter A1 (ABCA1 CEC) of cyclic-3′,5′-AMP (CTP-cAMP, Cayman Chemical Company, Ann Arbor, MI, USA) to induce ABCA1 expression. Efflux media containing 2.8% apoB-depleted serum was added for 4 h. The CEC of HDL was calculated as the percentage of radioactivity recovered from the medium to total radioactivity count. Three types of CECs were finally normalized with the CEC value of pooled control serum collected from healthy volunteers (age range, 27–32 years old, n = 4) on each plate to be presented as CEC indexes. ABCA1 CEC was calculated by subtracting the Non-ABCA1 CEC from Global CEC.

### Measurement of cholesterol levels in HDL subfractions

HDL subfractions were isolated from stored plasma by isopycnic density gradient ultracentrifugation with an Optima LE-80 K ultracentrifuge and 70.1 Ti rotor (Beckman Coulter, Palo Alto, CA, USA) with modification^40,41^. First, plasma was mixed with KBr solution to adjust the density to 1.063 g/mL and then ultracentrifuged at 40,000 rpm, for 24 h at 16 °C to float the fraction corresponding to VLDL and LDL. After that infranatant fluid was adjusted to 1.120 g/mL with KBr solution then ultracentrifuged at 40,000 rpm for 48 h at 16 °C to isolate HDL2 (d 1.063–1.120 g/mL). Finally, the infranatant fluid was adjusted to 1.210 g/mL and then ultracentrifuged at 40,000 rpm for 48 h at 16 °C to isolate HDL3 (d 1.120–1.210 g/mL). HDL2-C and HDL3-C levels were measured with LabAssay Cholesterol kit (FUJIFILM Wako Pure Chemical Corporation, Osaka, Japan).

### Statistical analysis

Continuous variables are presented as the mean ± standard deviation or median with interquartile range (IQR) in accordance with the results of the Shapiro–Wilk normality test, and were compared using the non-parametric Mann–Whitney test. Categorical data are shown as frequencies and percentages and were compared using the Fisher exact test followed by the chi-squared test. We performed adjusted multivariate logistic regression analyses by using three different models to assess the association between each of Global CEC, ABCA1 CEC, and Non-ABCA1 CEC of HDL, as explanatory variables, and the extent of atrial structural remodeling represented by enlarged LA (LAVI ≥ 34 mL/m^2^), as an objective variable. Variables used in models of multivariate analyses were identified by univariate logistic regression analyses, which were significantly associated with LAVI ≥ 34 mL/m^2^, in addition to age and gender. These three continuous variables, such as triglycerides, NT-proBNP and MPO, were substituted as logarithmically transformed (Log_10_) to the multivariate models, because of non-normal distribution. Furthermore, each CEC index, Model 1 of the adjusted multivariate analysis included age (1 year older), male gender, history of hypertension, smoking habit, and 1SD higher in LVEF and 1SD higher CEC indexes of HDL, respectively. Model 2 included the same variables as Model 1, with the exception of 1 higher Log_10_ NT-proBNP instead of 1SD higher of LVEF. Model 3 included 1 higher Log_10_ MPO and 1SD higher serum HDL-C in addition to all covariates in Model 1. Model 4 included all previously established risk factors for atrial structural remodeling in AF patients available in the present study, including age, male gender, hypertension, diabetes, smoking history, and biomarkers of heart failure (NT-proBNP), inflammation (hs-CRP) and obesity (BMI). All *p* values were two-tailed and considered as significant if less than 0.05.

### Ethical approval

This study protocol was approved by the Institutional Review Board of Juntendo University School of Medicine (IRB number 20–124).

## Supplementary Information

Supplementary Information 1.

## Abbreviations

AF: Atrial fibrillation
LA: Left atrium
LAVI: Left atrium volume index
LV: Left ventricle
HDL: High density lipoprotein
HDL-C: High density lipoprotein cholesterol
LDL: Low density lipoprotein
LDL-C: Low density lipoprotein cholesterol
ApoA1: Apolipoprotein A1
ApoB100: Apolipoprotein B100
CEC: Cholesterol efflux capacity
ATP: Adenosine triphosphate
ABCA1: ATP-binding cassette transporter A1
CETP: Cholesterol ester transfer protein
MPO: Myeloperoxidase
ELISA: Enzyme-linked immunosorbent assay
1SD: One standard deviation
