# Supplementary material for: The association between impairment of HDL cholesterol efflux capacity and atrial remodeling in atrial fibrillation
Source: Sci Rep. 2021 Feb 11;11:3547. doi: 10.1038/s41598-021-82998-4 (PMC7878912; doi:10.1038/s41598-021-82998-4)
Supplement: Supplementary file 1 — Supplementary Information 1. [file 41598_2021_82998_MOESM1_ESM.docx]

**Supplementary Materials:**

**Title:**

**The association between impairment of HDL cholesterol efflux capacity and atrial remodeling in atrial fibrillation**

**Authors:**

Asuka Minami-Takano, PhD^1,2^, Hiroshi Iwata, MD, PhD^1^, Katsutoshi Miyosawa, MS^1,3^, Tomoyuki Shiozawa, MD, PhD^1^, Hidemori Hayashi, MD, PhD^1^, Takehiro Funamizu, MD, PhD^1^, Kai Ishii, MD^1^, Yui Nozaki, MD^1^, Haruna Tabuchi, MD, PhD^1^, Gaku Sekita, MD, PhD^1^, Kazunori Shimada, MD^1^, Masataka Sumiyoshi, MD, PhD^4^, Yuji Nakazato, MD, PhD^5^, Hiroyuki Daida, MD^1^, Tohru Minamino, MD, PhD^1,6^

**Supplementary Tables**

**Supplemental Table 1: Univariate logistic regression analysis for large LAVI>34ml/m^2^**

|  | Odds Ratio | 95% Confidence Interval | *p* value |
| --- | --- | --- | --- |
| Age, 1 year older | 1.03 | (0.99-1.06) | 0.16 |
| Gender, male | 1.32 | (0.67-2.53) | 0.42 |
| Body mass index, 1 SD^1^ higher | 1.09 | (0.80-1.49) | 0.58 |
| **Hypertension** | **2.05** | **(1.09-3.94)** | **0.025** |
| Dyslipidemia | 1.20 | (0.65-2.21) | 0.56 |
| Diabetes | 2.37 | (0.93-7.31) | 0.074 |
| Chronic kidney disease | 1.46 | (0.62-3.87) | 0.40 |
| **Smoking history** | **1.87** | **(1.01-3.48)** | **0.045** |
| AF^2^ duration, 1 month longer | 1.57 | (0.89-2.82) | 0.12 |
| **Log_10_ Plasma NT-pro BNP^3^, 1 higher** | **9.85** | **(4.70-22.8)** | **<0.001** |
| Log_10_ Serum hs-CRP^4^, 1 higher | 1.15 | (0.82-1.59) | 0.42 |
| **LVEF^5^, 1 SD higher** | **0.56** | **(0.37-0.80)** | **0.001** |
| Log_10_ Triglycerides, 1 higher | 1.82 | (0.35-9.84) | 0.48 |
| LDL-C^6^, 1 SD higher | 0.91 | (0.66-1.25) | 0.58 |
| HDL-C^7^, 1 SD higher | 0.77 | (0.56-1.05) | 0.094 |
| ApoA1^8^-concentration, 1 SD higher | 0.79 | (0.57-1.07) | 0.15 |
| **Log_10_ Serum Myeloperoxidase, 1 higher** | **6.14** | **(1.422-29.30)** | **0.015** |
| **Global CEC^9^ of HDL, 1 SD higher** | **0.72** | **(0.52-0.98)** | **0.038** |
| ABCA1 CEC of HDL, 1SD higher | 0.93 | (0.70-1.26) | 0.66 |
| **Non-ABCA1^10^ CEC of HDL, 1 SD higher** | **0.70** | **(0.51-0.95)** | **0.021** |

1: standard deviation, 2: atrial fibrillation, 3: N-terminal pro b-type natriuretic peptide, 4: high-sensitivity C-reactive protein, 5: left ventricular ejection fraction, 6: low-density lipoprotein cholesterol, 7: high-density lipoprotein cholesterol, 8: apolipoprotein A1, 9: Cholesterol efflux capacity, 10: ATP-binding cassette transporter A1

**Supplementary Table 2**

**a Multivariate logistic regression analyses of Global CEC of HDL**

| Model 1 |  |  |  |
| --- | --- | --- | --- |
|  | Odds Ratio | 95% Confidence Interval | p value |
| Age, a year older | 1.03 | (0.98-1.07) | 0.17 |
| Gender, male | 0.98 | (0.43-2.20) | 0.96 |
| **Hypertension** | **2.17** | **(1.09-4.42)** | **0.027** |
| Smoking history | 1.64 | (0.79-3.42) | 0.18 |
| LVEF, 1 SD* higher | 0.51 | (0.32-0.75) | <0.001 |
| **Global CEC, 1 SD higher** | **0.7** | **(0.49-0.97)** | **0.032** |
|  |  |  |  |
| Model 2 |  |  |  |
|  | Odds Ratio | 95% Confidence Interval | p value |
| Age, a year older | 1 | (0.95-1.05) | 0.9 |
| Gender, male | 1.85 | (0.75-4.57) | 0.18 |
| **Hypertension** | **2.29** | **(1.08-5.05)** | **0.031** |
| Smoking history | 1.03 | (0.45-2.29) | 0.95 |
| **Log_10_ NT-proBNP, 1 higher** | **11.5** | **(5.06-29.15)** | **<0.001** |
| Global CEC, 1 SD higher | 0.69 | (0.48-1.00) | 0.052 |
|  |  |  |  |
| Model 3 |  |  |  |
|  | Odds Ratio | 95% Confidence Interval | p value |
| **Age, a year older** | **1.05** | **(1.00-1.10)** | **0.034** |
| Gender, male | 1.07 | (0.45-2.53) | 0.88 |
| **Hypertension** | **2.05** | **(1.01-4.25)** | **0.046** |
| Smoking history | 1.6 | (0.75-3.44) | 0.22 |
| **LVEF, 1 SD higher** | **0.54** | **(0.34-0.81)** | **0.0021** |
| HDL-C, 1 SD higher | 1.09 | (0.73-1.67) | 0.67 |
| **Log_10_ MPO, 1 higher** | **10.6** | **(1.89-68.30)** | **0.0068** |
| **Global CEC, 1 SD higher** | **0.66** | **(0.44-0.98)** | **0.039** |

* SD: standard deviation

**b Multivariate logistic regression analyses of ABCA1 CEC**

| Model 1 |  |  |  |
| --- | --- | --- | --- |
|  | Odds Ratio | 95% Confidence Interval | p value |
| Age, a year older | 1.03 | (0.98-1.07) | 0.2 |
| Gender, male | 1.02 | (0.45-2.27) | 0.95 |
| **Hypertension** | **2.13** | **(1.08-4.32)** | **0.03** |
| Smoking history | 1.57 | (0.76-3.24) | 0.21 |
| LVEF, 1 SD higher | 0.51 | (0.33-0.76) | <0.001 |
| ABCA1 CEC of HDL, 1 SD higher | 0.91 | (0.64-1.28) | 0.57 |
|  |  |  |  |
| Model 2 |  |  |  |
|  | Odds Ratio | 95% Confidence Interval | p value |
| Age, a year older | 1 | (0.96-1.05) | 0.91 |
| Gender, male | 1.94 | (0.80-4.78) | 0.14 |
| **Hypertension** | **2.29** | **(1.08-5.01)** | **0.031** |
| Smoking history | 0.97 | (0.43-2.14) | 0.95 |
| **Log_10_ NT-proBNP, 1 higher** | **11.25** | **(5.03-28.09)** | **<0.001** |
| ABCA1 CEC of HDL, 1 SD higher | 0.88 | (0.59-1.29) | 0.51 |
|  |  |  |  |
| Model 3 |  |  |  |
|  | Odds Ratio | 95% Confidence Interval | p value |
| **Age, a year older** | **1.05** | **(1.00-1.10)** | **0.036** |
| Gender, male | 1.01 | (0.43-2.36) | 0.97 |
| Hypertension | 2 | (0.99-4.11) | 0.053 |
| Smoking history | 1.58 | (0.75-3.34) | 0.23 |
| **LVEF, 1 SD higher** | **0.58** | **(0.36-0.83)** | **0.0032** |
| HDL-C, 1 SD higher | 0.88 | (0.62-1.27) | 0.49 |
| **Log_10_ MPO, 1 higher** | **9.6** | **(1.73-60.37)** | **0.0091** |
| ABCA1 CEC of HDL, 1 SD higher | 0.91 | (0.64-1.31) | 0.62 |

**c Multivariate logistic regression analyses of non-ABCA1 CEC**

| Model 1 |  |  |  |
| --- | --- | --- | --- |
|  | Odds Ratio | 95% Confidence Interval | p value |
| Age, a year older | 1.03 | (0.99-1.07) | 0.15 |
| Gender, male | 0.98 | (0.43-2.20) | 0.95 |
| **Hypertension** | **2.08** | **(1.05-4.24)** | **0.037** |
| Smoking history | 1.65 | (0.79-3.45) | 0.17 |
| LVEF, 1 SD higher | 0.61 | (0.33-0.76) | <0.001 |
| **Non-ABCA1 CEC of HDL, 1 SD higher** | **0.7** | **(0.48-0.94)** | **0.02** |
|  |  |  |  |
| Model 2 |  |  |  |
|  | Odds Ratio | 95% Confidence Interval | p value |
| Age, a year older | 1 | (0.95-1.05) | 0.87 |
| Gender, male | 1.78 | (0.73-4.42) | 0.21 |
| **Hypertension** | **2.25** | **(1.05-4.97)** | **0.035** |
| Smoking history | 1.03 | (0.46-2.29) | 0.94 |
| **Log_10_ NT-proBNP, 1 higher** | **11.29** | **(4.97-28.63)** | **<0.001** |
| **Non-ABCA1 CEC of HDL, 1 SD higher** | **0.69** | **(0.47-0.98)** | **0.042** |
|  |  |  |  |
| Model 3 |  |  |  |
|  | Odds Ratio | 95% Confidence Interval | p value |
| **Age, a year older** | **1.05** | **(1.01-1.10)** | **0.029** |
| Gender, male | 1.07 | (0.45-2.53) | 0.88 |
| Hypertension | 1.96 | (0.97-4.07) | 0.061 |
| Smoking history | 1.58 | (0.74-3.93) | 0.24 |
| **LVEF, 1 SD higher** | **0.54** | **(0.35-0.81)** | **0.0025** |
| HDL-C, 1 SD higher | 1.11 | (0.74-1.68) | 0.63 |
| **Log_10_ MPO, 1 higher** | **10.88** | **(1.92-70.62)** | **0.0064** |
| **Non-ABCA1 CEC of HDL, 1 SD higher** | **0.64** | **(0.43-0.95)** | **0.027** |

**Supplementary Figures**

**Supplementary Figure 1: Global CEC, ABCA1 CEC and Non-ABCA1 CEC in patients with paroxysmal and persistent atrial fibrillation (AF)**

**
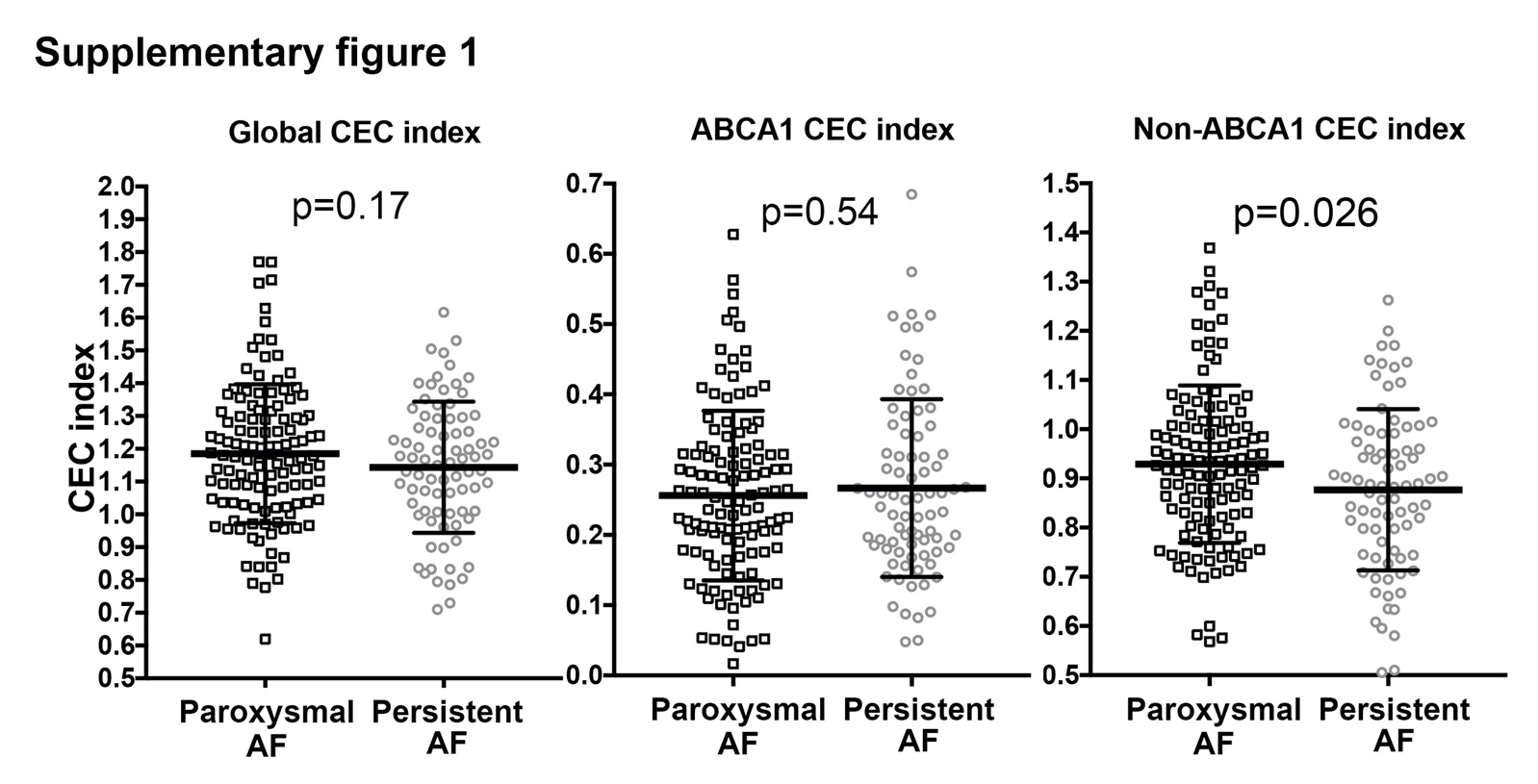
**

Global CEC, ABCA1 CEC and non-ABCA1 CEC were measured in AF patients with LAVI ≥ and < 34 mL/m^2^. The value was normalized with the respective QC value on each assay plate to calculate the indexed value. Each square/circle represents one participant. The horizontal lines indicate average ± standard deviation.

**Supplementary Figure 2: Global CEC, ABCA1 CEC and Non-ABCA1 CEC in age- and sex-matched Non-AF and AF patients who underwent catheter ablation**

**­­­
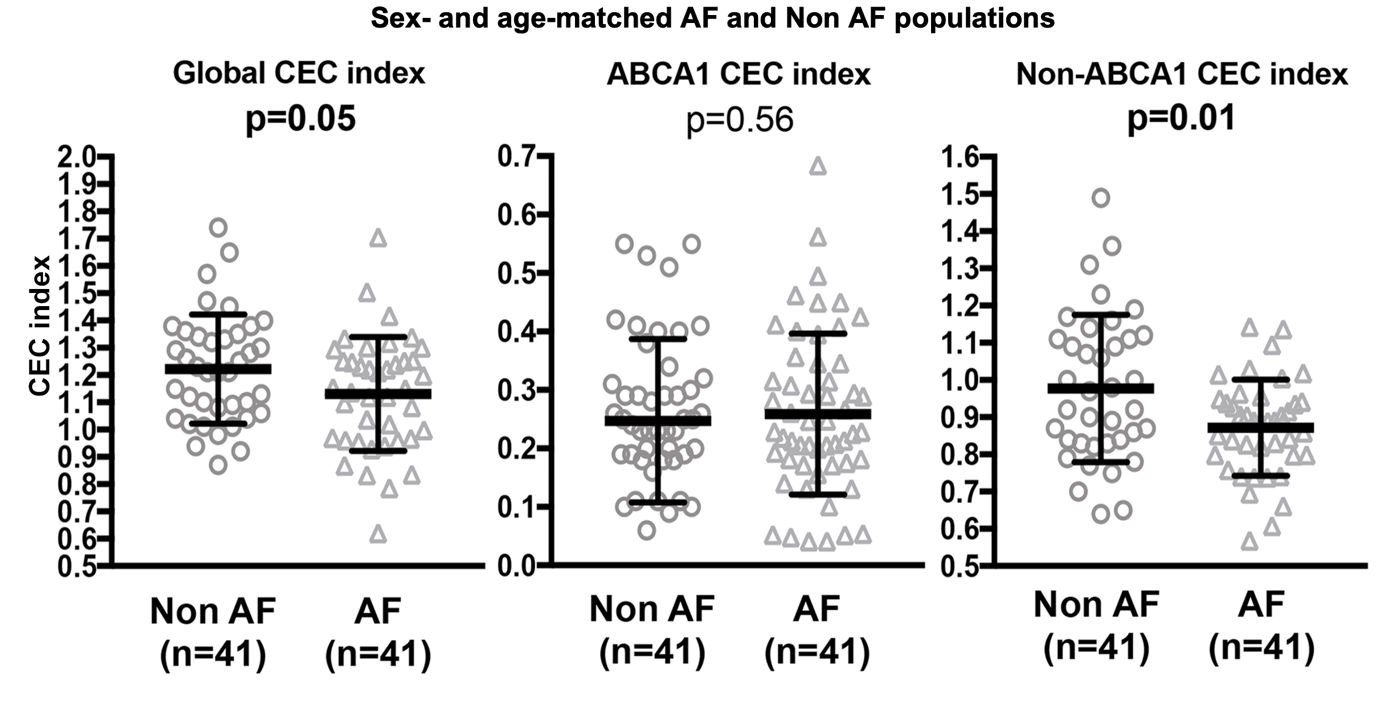
**

Each open circle/triangle represents one participant. The horizontal lines indicate average ± standard deviation.

**Supplementary Figure 3: Serum amyloid A (SAA) levels in patients with and without an enlarged left atrium (LAVI ≥ and < 34 mL/m^2^)**


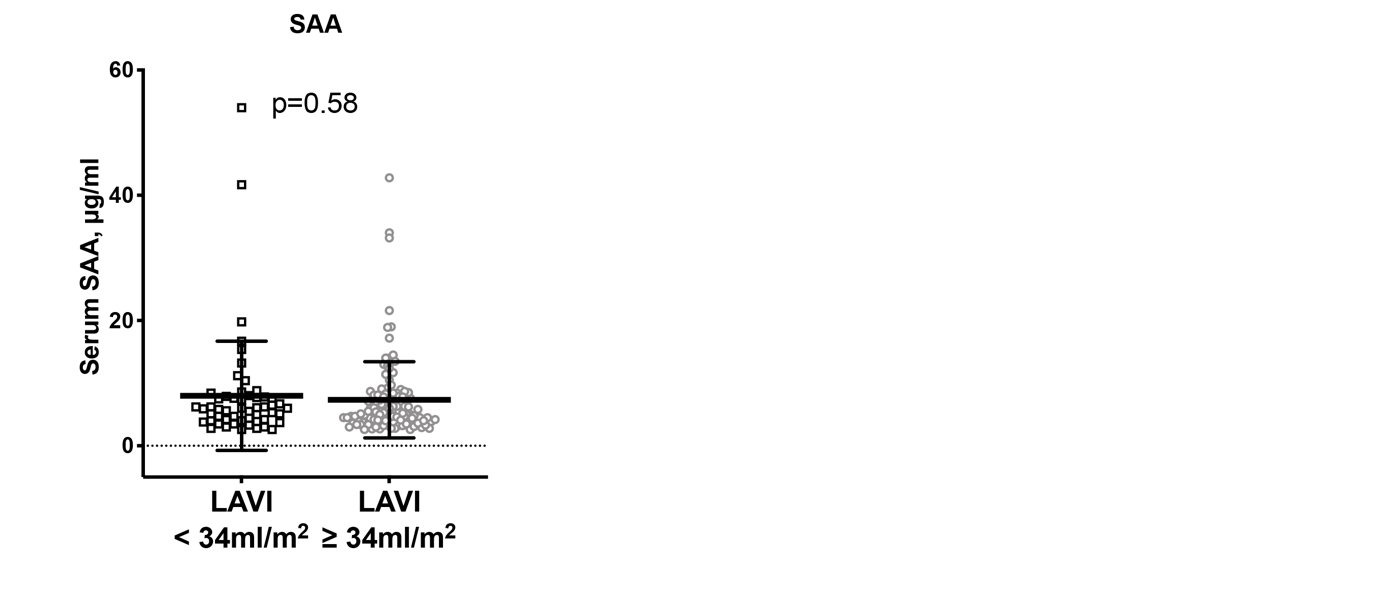


SAA levels were measured by latex immunoturbidimetric assay. Each open square/circle represents one participant. The horizontal lines indicate average ± standard deviation, respectively.

**Supplementary Figure 4: Correlation matrix in SAA, MPO, hs-CRP, LAVI and Global, Non-ABCA1 and ABCA1 CEC indexes**

**
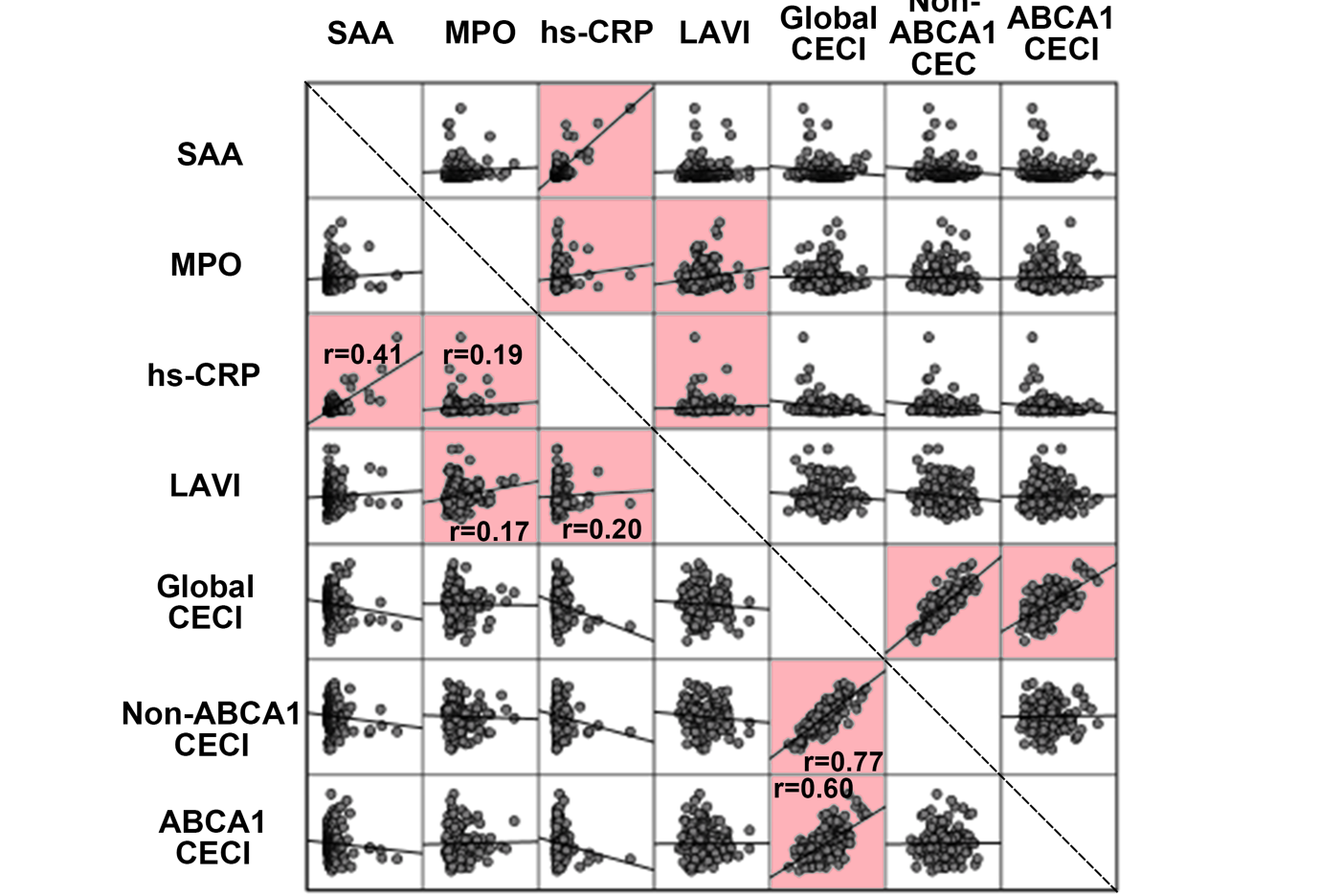
**

Each circle and the black line in each panel represents one participant and the regression line, respectively. Panels filled with red indicate a significant correlation between parameters in the rows and columns.

**Supplementary Figure 5: Consort diagram of this study**
